# Voxel Extraction and Multiclass Classification of Identified Brain Regions across Various Stages of Alzheimer’s Disease Using Machine Learning Approaches

**DOI:** 10.3390/diagnostics13182871

**Published:** 2023-09-07

**Authors:** Samra Shahzadi, Naveed Anwer Butt, Muhammad Usman Sana, Iñaki Elío Pascual, Mercedes Briones Urbano, Isabel de la Torre Díez, Imran Ashraf

**Affiliations:** 1 Department of Computer Science, Faculty of Computing and Information Technology, University of Gujrat, Gujrat 50700, Pakistan; samrashahzadi05@gmail.com (S.S.); naveed@uog.edu.pk (N.A.B.); 2 Department of Information Technology, University of Gujrat, Gujrat 50700, Pakistan; m.usman@uog.edu.pk; 3 Universidad Europea del Atlántico, Isabel Torres 21, 39011 Santander, Spain; inaki.elio@uneatlantico.es (I.E.P.); mercedes.briones@uneatlantico.es (M.B.U.); 4 Universidade Internacional do Cuanza, Cuito EN250, Bié, Angola; 5 Fundación Universitaria Internacional de Colombia, Bogotá 11001, Colombia; 6 Universidad Internacional Iberoamericana, Campeche 24560, Mexico; 7 Universidad Internacional Iberoamericana, Arecibo, PR 00613, USA; 8 Department of Signal Theory, Communications and Telematics Engineering, Unviersity of Valladolid, Paseo de Belén, 15, 47011 Valladolid, Spain; 9 Department of Information and Communication Engineering, Yeungnam University, Gyeongsan 38541, Republic of Korea

**Keywords:** Alzheimer’s disease detection, classification, machine learning

## Abstract

This study sought to investigate how different brain regions are affected by Alzheimer’s disease (AD) at various phases of the disease, using independent component analysis (ICA). The study examines six regions in the mild cognitive impairment (MCI) stage, four in the early stage of Alzheimer’s disease (AD), six in the moderate stage, and six in the severe stage. The precuneus, cuneus, middle frontal gyri, calcarine cortex, superior medial frontal gyri, and superior frontal gyri were the areas impacted at all phases. A general linear model (GLM) is used to extract the voxels of the previously mentioned regions. The resting fMRI data for 18 AD patients who had advanced from MCI to stage 3 of the disease were obtained from the ADNI public source database. The subjects include eight women and ten men. The voxel dataset is used to train and test ten machine learning algorithms to categorize the MCI, mild, moderate, and severe stages of Alzheimer’s disease. The accuracy, recall, precision, and F1 score were used as conventional scoring measures to evaluate the classification outcomes. AdaBoost fared better than the other algorithms and obtained a phenomenal accuracy of 98.61%, precision of 99.00%, and recall and F1 scores of 98.00% each.

## 1. Introduction and Background

Alzheimer’s disease (AD) is a neurological disease that slowly destroys brain cells, resulting in reduced memory, cognitive abilities, and daily function. It is a complicated illness that progresses gradually. This leads to brain cell death, which gradually impairs an individual’s capacity to complete tasks and causes loss of memory and thought [1]. Dementia is the result of the disease’s effects on cognitive function. One example of a neurodegenerative illness that not only affects localized grey matter but also results in improper integration across different sections of the brain is Alzheimer’s disease [2].

Presently, around 90 million individuals are affected by AD, and projections indicate that by 2050, the number of AD patients will be increased to 300 million [3]. Alzheimer’s disease develops in three stages, beginning with showing some symptoms of memory impairment, called mild cognitive impairment (MCI). As it progresses to each subsequent stage, the disease becomes more severe, and patients experience difficulty performing everyday tasks. Studies [4,5,6,7] have conducted research to determine which particular parts of the brain are impacted by AD. The study [8] conducted research on AD to explore those aspects of AD which are not covered by the previous literature. As a result, the goal of their groundbreaking research is to examine the afflicted brain areas at each stage of AD. They also classify AD patients into three groups based on their MCI status: mild, moderate, and severe.

The study [8] conducted research on AD to explore those aspects of AD which are not covered by the previous literature. Therefore, the objective of the research, which is novel, is to analyze the affected brain regions during each stage of AD. The authors also perform binary classification between MCI and mild, MCI and moderate, and MCI and severe stage of AD patients. Similarly, ref. [9] used a support vector machine (SVM) learning algorithm to distinguish AD from healthy control (HC) and MCI from HC by considering data from 52 HCs patients, 99 MCI patients, and 51 AD patients. Alzheimer’s disease was classified with 93.2 percent accuracy in healthy controls, while mild cognitive impairment was classified with 76.4 percent accuracy in healthy controls.

The authors used classical classification methods SVM, K-near neighbor (KNN), and Naive Bayes (NB) to classify healthy and AD patients in [10]. The data include brain scans from 416 people aged between 18 and 96 years. The analysis showed that the SVM could classify normal brains from Alzheimer’s brains with 95 percent accuracy. NB and KNN, on the other hand, delivered 90 percent accuracy for the data used for experiments.

The authors in [11] observed during their research that Alzheimer’s disease affects the entire brain and that the regions with the most significant differences are the posterior cingulate cortex (PCC), middle temporal gyrus (MTG), entorhinal cortex, and hippocampus. The author employed machine learning methods SVM, linear discriminant analysis (LDA), artificial neural network (ANN), and logistic regression (LR) to classify AD stages using resting-state fMRI data. The authors considered three stages of AD, including mild, moderate, and severe, and used machine learning algorithms to identify different stages of AD.

The study [12] examines various machine learning architectures to distinguish AD from mild cognitive impairment (MCI). DenseNet-169 emerges as the top performer, achieving an impressive 82.2% accuracy in AD classification from MCI. Similarly, the authors explore health records using manifold learning techniques to differentiate early-stage AD groups [13]. Various methods, such as spectral embedding and autoencoder-based manifolds, are applied to the ADNI dataset for insightful analysis.

In [14], a pioneering deep learning model known as FDN-ADNet is introduced. The model aims to enhance early AD diagnosis by extracting features across hierarchical levels. It employs a Fuzzy Hyperplane-based least square twin support vector machine (FLS-TWSVM) classification technique and uses sagittal plane slices from 3D MRI images to train the FDN-ADNet model. With impressive classification accuracy rates of 97.15% for CN vs. AD, 97.29% for CN vs. MCI, and 95% for AD vs. MCI, the model excels in distinguishing cognitive states. This approach shows potential as a robust tool for early AD diagnosis.

Sadiq et al. [15] introduced an automated framework that addresses existing limitations through a comprehensive exploration of signal decomposition, feature selection, and neural network methods. By achieving substantial enhancements of up to 26.1% and 26.4% compared to current state-of-the-art approaches, this framework holds potential for both subject-dependent and independent brain–computer interface (BCI) systems. It offers a pathway for the creation of adaptable BCI devices, catering to motor-disabled users’ interaction needs. Similarly, ref. [16] introduces an innovative automated computerized framework to effectively detect motor and mental imagery (MeI) EEG tasks. The approach leverages empirical Fourier decomposition (EFD) and improved EFD (IEFD) techniques. The EEG data are initially denoised using multiscale principal component analysis (MSPCA), followed by EFD to decompose nonstationary EEG into modes. The IEFD criterion facilitates the selection of a distinct mode. Time- and frequency-domain features are then extracted and classified using a feedforward neural network (FFNN) classifier.

The study [17] utilizes the multivariate variational mode decomposition (MVMD) on 18-channel EEG data from the motor cortex, along with the Relief-F feature selection technique, yielding remarkable results. In subject-dependent experiments, the approach achieved an average classification accuracy of 99.8%. For subject-independent experiments, the accuracy reached 98.3%. Moreover, this combination consistently delivered accuracy exceeding 99% for subjects with either ample or limited training samples, across both subject-dependent and independent scenarios. These impressive outcomes underscore the framework’s adaptability for subject-specific and subject-independent BCI systems.

The authors proposed a framework in [18] which consists of three main phases: firstly, investigating the chaotic nature of EEG signals through phase space dynamics visualization; secondly, extracting thirty-four graphical features to decode chaotic patterns in normal and alcoholic EEG signals; thirdly, selecting optimal features using various techniques combined with machine learning and neural network classifiers. Results show that the method achieves remarkable classification performance, with 99.16% accuracy, 100% sensitivity, and 98.36% specificity. The combination of Henry gas solubility optimization and feedforward neural network stands out as the most effective approach.

The study [19] employs multiscale principal component analysis for robust noise reduction in the preprocessing phase. A novel automated strategy for channel selection is introduced and validated through comprehensive comparisons of decoding methods. The study pioneers the use of MEWT to capture joint amplitude and frequency components, enhancing Motor Imagery applications. The study applies a robust correlation-based feature selection technique to reduce system complexity and computational demands.

Akbari et al. [20] introduces a novel strategy to diagnose depression using geometric features extracted from EEG signal shapes via second-order differential plots (SODPs). Various geometric characteristics are derived from the SODPs of normal and depressed EEG signals. Binary particle swarm optimization selects relevant features, which are then used with SVM and k-nearest neighbor classifiers to identify depression accurately. This approach capitalizes on the distinctive geometric properties of EEG signals to improve depression diagnosis. Similarly, ref. [21] proposes novel geometric features for distinguishing between seizure (S) and seizure-free (SF) EEG signals. The features are derived from the Poincaré pattern of discrete wavelet transform coefficients, utilizing distinct plot patterns. These features, including 2D projection descriptors (STD), triangle area summation (STA), the shortest distance from the 45-degree line (SSHD), and the distance from the coordinate center (SDTC), aid in classifying EEG signals accurately.

The study [22] used a Gaussian process (GP) machine learning algorithm to classify MCI and AD. The authors applied the GP model to 77 subjects, 50 MKI and 27 AD. All subjects had resting-state fMRI data. Experimental results demonstrate a 97 percent accuracy for GP and observed that GP performs effectively in MCI and AD classification. Ref. [23] used SVM, LDA, and logistic regression (LR) to classify susceptible brain regions associated with Alzheimer’s disease. They used predefined regions of the AD hippocampus, MTG, PCC, and entorhinal cortex. They did not define regions with independent component analysis (ICA). The study’s goal was to identify brain areas implicated in various phases of Alzheimer’s disease. Independent component analysis (ICA) was used to extract information from mixed data from fMRI data in order to localize specific brain areas affected by distinct stages of AD. The author used SPM12 to extract voxel data for the associated brain areas at each stage when applying ICA to specify brain regions. SPM12 implements a generalized linear model (GLM) applied to each voxel of a functional image. The voxel data extracted in the previous step were further used for multi-class classification of the stages using machine learning algorithms.

In contrast to earlier studies, the goal of this study is to investigate the regions of the brain that are impaired at each stage of the formation of AD. Six affected brain regions with moderate cognitive impairment were examined, four in the preliminary stages of AD and six each in the subsequent stages. The precuneus, cuneus, middle frontal gyri, calcarine cortex, superior medial frontal gyri, and superior frontal gyri were common brain areas affected by all these stages. The brain activations vary among all these stages. Brain activation reduces as the stages progress.

Functional change in one patient varies from one stage to the other. If the patient data have changed in each stage, then it is not possible to observe the functional changes in all four stages. To observe the functional changes in all four stages, the data include brain scans from the same 18 individuals for all four stages. The voxels have been extracted from the six affected brain regions. These regions were defined by the regional changes during all four stages. Data from the same patients for all four stages are not available.

This study used four stages by including healthy diseases of the same patients for multi-class classification. To the best of our knowledge, we are the first to classify our dataset into multiple stages with the selected specific regions using machine learning models. The healthy stage, the early stage, and the end stage of AD were the main classification pillars. For the multi-class classification, we selected the same patients in the four phases of AD.

Despite the contribution of the above-discussed studies, this study still lacks high-accuracy models. Machine learning models are not very well investigated for Alzheimer’s disease regarding their use at different levels of AD and the influence of AD on different regions of the brain. In this regard, this study applies eight machine learning models, both linear and nonlinear, which include neural networks, linear SVM, polynomial SVM, nearest neighbors, decision trees (DT), random forests (RF), AdaBoost, and NB. The primary objective is to find specific brain regions affected at any stage of AD based on fMRI data. To extract brain morphological patterns, all machine learning algorithms successfully classified AD patients using data from selected regions with a satisfactory classification accuracy of more than 90%. Experimental results show that AdaBoost and KNN techniques achieve high classification accuracy (98%) and perform well on this particular dataset.

This study is further divided into three sections. Section 2 presents the proposed methodology and explains different phases of the approach adopted for experiments. Results and analysis are presented in Section 3, while Section 4 provides discussions. In the end, Section 5 concludes this study.

## 2. Materials and Methods

### 2.1. Dataset Description

The Alzheimer’s Disease Neuroimaging Initiative’s ADNI dataset (https://adni.loni.usc.edu Accessed on 21 April 2023) was used in this investigation. ADNI is a multicenter study that began in 2004 as a private–public collaboration between 20 companies and two foundations, the National Institute on Aging and the National Institute of Health, and is led by Michael W. Weiner. The ADNI intervention seeks to detect Alzheimer’s disease at an early stage and track its progression. The ADNI project gathered information from fMRI, MRI, PET, and other diagnostic methods used to track the progression of MCI and AD. Our research was divided into four phases: ADNI-1, ADNI-Go, ADNI-2, and ADNI-3. We analyzed 18 individuals with Alzheimer’s disease who were migrating from moderate cognitive impairment to mild Alzheimer’s disease using resting-state fMRI data from the ADNI public resource database. There were eight (8) females and ten (10) males in the study. Each patient is assigned a unique ID, which may be one of the following: 2036F, 2045M, 2055M, 2073F, 2123F, 2130M, 2133F, 2155F, 2180F, 2191F, 2195M, 2208M, 2225M, 2264M, 2274M, 2301M, 2304M, or 2373F, where ’F’ denotes a female and ’M’ denotes a male. The resting-state functional magnetic resonance imaging was created using a Philips scanner; each individual underwent 105 scans; the volume size was X = 64, Y = 64, and Z = 20; the voxel size was X = 4, Y = 4, and Z = 5.3T; and the repetition time (TR) was 3400.0 milliseconds.

Over a 12-year period, data on the same 18 AD patients in each of the stages of the disease were gathered [8]. Stage 1 data were gathered for the first four years, Stage 2 data for the next four years, and Stage 3 data were gathered in the same manner for the patient’s final four years. For each AD patient at each stage, 105 volumes were chosen, and calculations were performed on 1890 volumes at each stage, as shown in Table 1. The only regions chosen are those on the left and right.

### 2.2. Methodology

**Figure 1:**
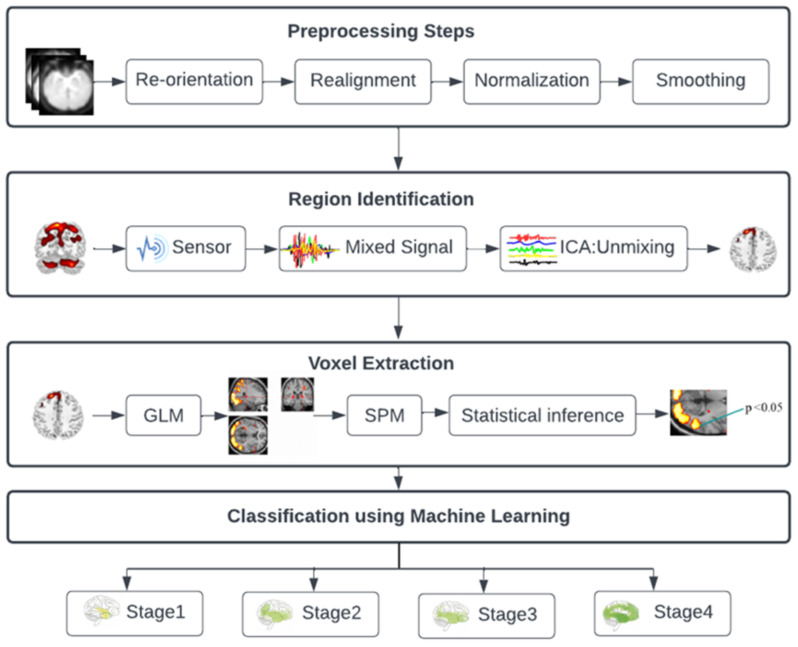
Diagram for the architecture of the proposed approach.

Figure 1 illustrates the four steps of the suggested approach. In the first stage, mixed data information was extracted from the fMRI data using ICA, allowing the localization of specific brain regions affected at different stages of AD. In the second stage, a machine learning technique is used to separate the three stages of MCI from AD. These phases are described in the parts that come after. Voxel extraction is the third stage, followed by classification in the fourth.

#### 2.2.1. Phase 1: Preprocessing

Neuroimaging studies aim at understanding brain structure and function, and the choice of imaging modality, meticulous preprocessing pipelines, and robust quality control measures are paramount to ensure the validity and reliability of the obtained results. These elements collectively contribute to minimizing artifacts, noise, and bias, thereby enabling accurate interpretations of brain data. fMRI captures blood-oxygen-level-dependent (BOLD) signals, indicating brain activity. Preprocessing includes motion correction, slice timing correction, spatial normalization, and smoothing to enhance signal-to-noise ratio.

The data must first be cleansed using a variety of preprocessing processes before analysis can be performed. To preprocess the fMRI data, we utilized the SPM tool (SPM12 in Matlab 2022b), which involved four procedures: reorientation, realignment, normalization, and smoothing. This phase aimed at removing the effects of preprocessing from the data. First, the functional images were reoriented to align with the temple image “EPI.nii”, ensuring that each image could be compared to others. Next, we applied realignment, also known as motion correction, to further minimize the noise in the realigned data. A person’s head can tilt to the left or right, up or down, so this kind of correction is required. To remove these two sorts of noise, the ’Translation’ and ’Rotation’ must be rescaled. The functional pictures were changed to conform to typical MNI template brains using normalization. This makes it easier to compare the functional activation of different people. After completing normalization, we smoothed all the realigned and normalized images. Averaging data points with nearby points is the process of smoothing, which also entails hiding sharp edges. This process amplifies low-frequency signals while dampening high-frequency impulses.

#### 2.2.2. Phase 2: Region Identification

In order to pinpoint specific brain regions impacted by various stages of AD, information from the mixed data was extracted from the second-stage group’s fMRI data using independent component analysis (ICA). ICA, alternatively referred to as Blind Source Separation (BSS), is primarily employed to identify the independent hidden factors responsible for generating the information [24]. Additionally, it has been utilized for extracting components from fMRI data. Several existing studies [25,26,27,28] used ICA to analyze the fMRI data.

The study [8] used ICA to specify which areas of the brain are problematic at each stage of AD. For each patient at each stage, the mixed signals produced by various brain regions were separated by using ICA. This is accomplished with the aid of a “GIFT” toolset. The activation is moved away from the location where it was initially formed. The precise name of the site is not provided by GIFT, but it may identify an active zone and provide its MNI peak coordinates. Moreover, MRICron uses the provided aal.nii picture to identify particular locations using the GIFT-provided coordinates.

#### 2.2.3. Phase 3: Voxel Extraction

Statistical parameter mapping (SPM) is widely accepted within the neuroimaging community and is considered a standard method for analyzing neuroimaging data. Each voxel of a functional image is subjected to the general linear model (GLM) implemented by the software. The approach employed here is known as a “mass-univariate” method. It involves a design matrix that is uniform across all voxels, along with voxel-specific parameter estimates. The design matrix incorporates information related to the activation paradigm as well as any potential confounding factors. The parameter estimates signify the intensity of activations and confounds present in each voxel. After initial preprocessing steps, the data undergo spatial smoothing, and a GLM is applied to each voxel. Next, a statistic is calculated for each voxel to identify those that demonstrate significant activity. Due to the vast number of voxels involved, some may show activity by mere chance. To address this issue, a correction for multiple comparisons is carried out, utilizing Gaussian random field (GRF) theory.

#### 2.2.4. Phase 4: Classification

In the fourth phase, classification is completed using a variety of machine learning models. Here is a brief summary of each of the models that were used.

Linear SVM uses hyperplanes to divide data into classes. Two hyperplanes are built using the supervised machine learning method of SVM for linear classification. In general, increasing the space between hyperplanes during training increases the classifier’s accuracy during testing. The format of the number *N* of training cases is $\left(x_{1},y_{1}\right),\left(x_{2},y_{2}\right),\ldots ,\left(x_{n},y_{n}\right)$, where *y* is 1 or −1 and represents the category of *x*. When the feature space is very big in classification and regression issues, it produces exceptionally accurate results. SVM aids in determining the ideal course between potential outcomes. The highest level of accuracy is offered by its extremely effective algorithm, particularly in categorization. Also, it is a developed form of the linear classifier.

NB is a probabilistic classifier that determines the category of a certain occurrence using the Bayes theorem. It applies the conditional probability model using the inputs $X=(X_{1},X_{2},\ldots )T$ and assigns a probability to the instances in the manner specified [29]. The Bayesian theorem’s formula is provided below.

(1) $$P\left(C|X\right)=\frac{P(X|C)P(C)}{P(X)}$$

where P(C|X) is the posterior probability, P(X|C) is likelihood, P(C) is class prior probability, P(X) is predictor prior probability, and *C* denotes the classes.

Like other quadratic classification methods, Quadratic Discriminant Analysis (QDA) is used for classification using quadratic decision surfaces: circular, ecliptic, parabolic, or hyperbolic. Compared to LDA, QDA is considered more powerful because it allows greater flexibility in the covariance matrix, relaxed under the assumption of covariance equality $\left(\sum_{1}=\sum_{2}\right)$, but increases the computational burden and parameters. The number of parameters increases because QDA has a separate covariance matrix for each class. In the case of bivariate classification, the class of a case *x* is estimated as

(2) $$y=\left\{\begin{array}{cc} 1, & \mathrm{if}\;\delta (x)<0\\ 2, & \mathrm{if}\;\delta (x)>0 \end{array}\right.$$

where

(3) $$\delta \left(x\right)=x^{T}{\left(\sum_{1}-\sum_{2}\right)}^{-1}x+2{\left(\sum_{2}^{-1}\mu_{2}-\sum_{1}^{-1}\mu_{1}\right)}^{T}x+\left(\mu_{1}^{T}\sum_{1}^{-1}\mu_{1}-\mu_{2}^{T}\sum_{2}^{-1}\mu_{2}\right)+ln\left(\frac{|\sum_{1}|}{|\sum_{2}|}\right)$$

where $\mu_{1}$ and $\mu_{2}$ are the mean vectors of classes 1 and 2, and $\sum_{1}$ and $\sum_{2}$ are corresponding covariance matrices of the first and second class.

A DT is a very powerful classification and prediction tool. Each internal node represents a test on an attribute, each branch shows the test results, and each terminal node has a class label. As such, it resembles a flowchart. The comparison of DT to other classification techniques is possible since DT is a form of supervised learning. The fundamental goal of employing DT is to build a training model for class prediction by learning decision-making processes from historical data. The literature contains extensive documentation of the methodology.

(4) $$X,Y=x_{1},x_{2},x_{3},\ldots ,x_{m}$$

where *Y* serves as the dependent variable, goal variable, and classification variable. *X* is a vector of independent variables for this task $\left(X=x_{1},x_{2},\ldots ,x_{m}\right)$.

In the context of a dataset, the notation (x,Y) represents pairs of data instances and their corresponding outcomes. *x* is a vector containing the features or attributes associated with a particular data instance. Each data instance is represented by a feature vector (x1,x2,x3,…,xk), where *k* represents the number of features. These features provide information that is used to predict the target variable *Y*. *Y* represents the dependent variable or target variable of interest. It is the outcome or value we are aiming to understand, classify, or predict based on the features *x*. The variable *Y* could take on different forms based on the nature of the problem. For instance, in a classification problem, *Y* might represent different classes or labels that we want to assign to data instances based on their features.

In summary, (x,Y) denotes the relationship between feature vectors *x* and their corresponding target variable *Y*. This relationship is leveraged in various analytical and machine learning tasks to gain insights, make predictions, or perform classifications.

KNN is a supervised machine learning technique that is utilized as a solution to the classification problem. This approach assumes that similar things are nearby. Thus, by considering this assumption, a distance-based separation of classes can be made. It is clear, concise, and involves a variety of functions. Behind the algorithm of KNN, the distances between data points are calculated and the decision is drawn based on these distances.

AdaBoost, or adaptive boosting, is a machine learning boosting algorithm that is often used for classification issues. This method is referred to as adaptive boosting because it involves reassigning weights to each example, with higher weights being assigned to inaccurately classified examples [30].

In AdaBoost, several weak learners (also called base learners) are constructed one after the other, and the error is calculated after every weak learner. If it is higher than the desired level, then the weights as adjusted and using the new weights are used by the next learner. Each weak learner consists of one node with two leaves, called stump, as shown in the figure above. We can see a weak learner that has only two nodes. The order of the weak learners is very important in this technique because the error of the first learner influences how the second learner is made. After arriving at a desired acceptable loss, these weak learners are combined with corresponding weights to make the final prediction. Mathematically, it can be expressed as

(5) $$G\left(x\right)=sign(\sum_{m=1}^{M}\alpha_{m}G_{m}\left(x\right))$$

where the contributions of each individual $G_{m}\left(x\right)$ are weighted and the numbers $\alpha_{1},\alpha_{2}\ldots ,\alpha_{M}$ are computed using the boosting algorithm.

RF is another powerful machine learning algorithm commonly used for classification purposes. It is built on the idea of ensemble learning, which entails fusing various classifiers or algorithms to produce a solitary, reliable separation tool. On several subsets of a given dataset, random forest creates many decision trees, taking the average to produce predictions. The random forest makes its predictions based on the consensus of numerous decision trees rather than depending solely on one. The more trees that are included, the more accurate the predictions and the less the likelihood of overfitting [31].

Artificial neural network (ANN), also known as multilayer perceptron, is a powerful technique used for pattern recognition and separation of objects. There are numerous layers in it, including the input layer, hidden layers, and the output layer. Every layer’s nodes are connected. By adding more hidden layers to the network, it can get deeper. The network can be made deeper by increasing the number of hidden layers in it. The $x_{1},x_{2},\ldots ,x_{n}$ are the inputs and $w_{1},w_{2},\ldots ,w_{n}$ are the associated weights, ∑ is the weighted sum, *f* is the activation function, and $y_{1}$ is the output. The information given to the network is passed through an activation function that provides an output. Mathematically, it can be represented as

(6) $$y=f\left(x.w\right)=f\left(\sum_{i=1}^{n}x_{i}.w_{i}\right)$$

If the bias term is included, then the above equation can be written as

(7) $$y=fb+\left(x.w\right)=f\left(b+\sum_{i=1}^{n}x_{i}.w_{i}\right)$$

The procedure described above is the forward pass. For better prediction and better weights, backpropagation can also be carried out. The steps that are performed in neural networks are listed below.

- Randomly initialize weights of all the input nodes. There are many methods available now to initialize weights.
- Train the model using the forward pass procedure described above and take outputs.
- Use the predicted classes and actual classes to calculate error/loss using a loss function.
- If the loss is greater than the desired value, perform the backward pass to update weights using a learning rate and then perform the forward pass using the updated weights.
- Continue to update weights until the desired loss value is achieved.

### 2.3. Training and Learning of Models

Choosing the right architecture and hyperparameters for machine learning algorithms is one of the most vital considerations. Details for hyperparameters used in this study are briefly discussed here.

#### 2.3.1. ANN

Generalized error (often called test error) must be kept as small as possible in all machine learning algorithms. The performance of a machine learning algorithm is affected by its ability to reduce training errors and the difference between training and testing errors. The algorithms were trained and validated using a train/test ratio of 0.8 to 0.2 and 10-fold cross-validation to achieve the lowest overall error.

#### 2.3.2. AdaBoost

AdaBoostClassifier is an ensemble learning algorithm that combines multiple weak classifiers to create a stronger overall classifier. Some changeable parameters are also set up, together with their permitted or fixed values. For experiments, max_depth = 2, n_estimators = 300, and learning_rate = 1. These parameters offer flexibility in tuning the AdaBoostClassifier according to this experiment domain and dataset.

The max_depth parameter specifies the base estimator used within the AdaBoost. In this case, a decision tree classifier is chosen as the weak learner. The max_depth parameter is set to 2, which limits the depth of each decision tree in the ensemble. Restricting the depth helps prevent overfitting and improves the model’s generalization ability.

The n_estimators parameter determines the number of weak learners (decision trees) to be combined in the ensemble. In this study, 300 decision trees are trained sequentially. Each subsequent tree focuses on correcting the mistakes made by the previous trees. Increasing the number of estimators can improve the classifier’s performance, but it can also increase computation time.

The learning_rate controls the contribution of each weak learner to the final ensemble. A learning rate of 1 means that each weak learner’s prediction is given full weight in the ensemble. The learning rate can be adjusted to control the impact of each weak learner’s contribution. Lower learning rates often require more estimators to achieve good performance.

#### 2.3.3. Decision Tree

The DT is a machine learning algorithm that builds a decision tree model to make predictions. Some changeable parameters are also set up, together with their permitted or fixed values. For experiments, entropy is used as ’criterion’, while max_depth and random_state are set to 5 and 0, respectively. These parameters offer control over the splitting criterion, depth, and randomness in the decision tree algorithm.

The criterion parameter specifies the criterion used for measuring the quality of a split. In this case, ’entropy’ is selected as the criterion. Entropy is a measure of impurity in a node, and the decision tree algorithm aims to minimize entropy by selecting features that best separate the classes in the data.

The max_depth determines the maximum depth of the decision tree. By setting it to 5, the tree will be grown to a maximum depth of 5 levels. Limiting the depth helps prevent overfitting, as a shallow tree tends to generalize better. Adjusting the max_depth value is important for finding the right balance between model complexity and generalization.

The random_state parameter sets the random seed for the random number generator. By providing a value of 0, we ensure that the algorithm’s randomness is reproducible. This means that running the algorithm with the same random_state value will produce the same results each time. Setting random_state can be helpful when consistent results are needed for debugging or comparison purposes.

#### 2.3.4. KNN

KNN is a machine learning algorithm that performs classification based on the k-nearest neighbors approach. For KNN, the n_neighbors is set to 5 while *P* is set to 1. These parameters allow us to control the behavior of the KNN algorithm.

The n_neighbors parameter determines the number of neighbors considered for classification. In this case, the algorithm classifies a new data point based on the labels of its five nearest neighbors. The choice of the number of neighbors depends on the nature of the dataset and the problem at hand. Higher values of n_neighbors smooth out the decision boundary but may oversimplify the model, while lower values can lead to more localized and potentially noisy decisions.

The metric parameter specifies the distance metric used to measure the proximity between data points. ’Minkowski’ is a generalization that includes various distance metrics, such as Euclidean distance and Manhattan distance. The specific metric used is based on the *p* parameter. The *p* parameter determines the power parameter for the Minkowski distance metric. When *p* = 1, the Minkowski distance becomes equivalent to the Manhattan distance, also known as the L1 norm. Alternatively, setting *p* = 2 corresponds to the Euclidean distance, known as the L2 norm. The choice of *p* depends on the characteristics of the data and the problem. The L1 norm may be suitable when dealing with sparse data, while the L2 norm is commonly used for continuous data.

#### 2.3.5. Linear SVM

SVM is a machine learning algorithm that is commonly used for classification problems. Let us discuss the parameters used in this study. A linear kernel is used in this study with C = 0.025, while probability is set to ’true’. These parameters provide flexibility in customizing the behavior of the SVM.

The kernel parameter specifies the type of kernel function to be used for the SVM. In this study, a linear kernel is chosen. A linear kernel creates a linear decision boundary and is suitable for linearly separable data.

The C parameter is the regularization parameter in the SVM algorithm. It controls the trade-off between maximizing the margin (finding a larger margin hyperplane) and minimizing the classification errors on the training data. Smaller values of C result in a wider margin but may lead to more misclassifications, while larger values of C prioritize accurate classification of training examples, potentially resulting in a narrower margin.

The probability parameter enables probability estimates in the SVM. When set to true, the algorithm uses Platt scaling to estimate class probabilities, allowing us to obtain probability estimates for each class prediction. Enabling probability estimates may increase training time and memory requirements.

#### 2.3.6. RBF SVM

For RBF SVM, kernel, degree, c, and probability parameters are fine-tuned. It is used with the ’Poly’ kernel, while the degree is set to 3. Probability is set to ’true’, while 1 is used as the C value.

The kernel is set to the polynomial kernel for RBF SVM. The polynomial kernel allows for non-linear decision boundaries by mapping the input features into a higher-dimensional space. It is particularly useful when the data are not linearly separable.

The degree parameter specifies the degree of the polynomial kernel function. In this study, a degree of 3 is selected, which means the polynomial kernel function is cubic. The degree determines the complexity of the polynomial mapping applied to the data. Higher degrees can capture more complex relationships between the features but may also increase the risk of overfitting. It is important to choose an appropriate degree based on the complexity of the problem and the available training data.

The C parameter is the regularization parameter in the SVM. It controls the trade-off between achieving a low training error and maximizing the margin.

#### 2.3.7. Gaussian NB

The GNB algorithm is a machine learning algorithm that implements the GNB classifier. The Gaussian Naive Bayes classifier is based on Bayes’ theorem and makes predictions by calculating the likelihood of each class given the observed features. It assumes that the features are conditionally independent, meaning that the presence or value of one feature does not affect the others given the class label. Despite this strong assumption, the GNB classifier can perform well in practice and is particularly useful when dealing with large feature spaces. One advantage of the GNB algorithm is its simplicity and efficiency. It does not require tuning hyperparameters or complex training procedures, making it computationally inexpensive.

#### 2.3.8. Neural Network

The NN is a machine learning algorithm that implements a multi-layer perceptron (MLP) neural network for classification. The NN is a versatile algorithm that can learn complex relationships between features and target labels. However, it is sensitive to various hyperparameters and requires careful tuning to achieve optimal performance. Some changeable parameters are also set up, together with their permitted or fixed values.

The random_state parameter sets the random seed for reproducibility. By specifying a specific value (in this case), we ensure that the random initialization of weights and biases in the neural network is consistent across different runs. Setting a random seed allows us to obtain the same results each time you train the model, which can be useful for debugging and result comparison purposes.

The max_iter parameter determines the maximum number of iterations or epochs that the classifier will perform during training. An epoch refers to a complete pass through the entire training dataset. By setting max_iter to 300, the training process is limited to a maximum of 300 epochs. Controlling the number of iterations is crucial to prevent overfitting, as training for too many epochs can lead to the model memorizing the training data.

#### 2.3.9. Random Forest

The RF is a machine learning algorithm that builds an ensemble of decision trees and combines their predictions to make classification decisions. These parameters control the behavior of the RF algorithm. Some changeable parameters are also set up, together with their permitted or fixed values.

The n_estimators parameter determines the number of decision trees in RF. Generally, increasing the number of estimators improves the performance of the random forest by reducing the impact of individual trees’ variability. However, using a smaller number of estimators can be useful for debugging or quickly assessing the model’s initial performance.

The max_features parameter controls the maximum number of features considered for each tree when searching for the best split. By setting it to None, the algorithm considers all features at each split. The bootstrap parameter determines whether bootstrap samples are used to train each decision tree. When set to false, the entire training dataset is used to build each tree.

The random_state sets the random seed for reproducibility. By providing a specific value (in this case, 1), the random processes involved in building the random forest are ensured. Setting a random seed allows us to obtain the same results each time you train the model, which is useful for debugging and result comparison.

Table 2 provides a summary of all the parameters used for models employed in this study.

## 3. Results and Analysis

After data preprocessing, we looked at the brain regions in MCI, including mild, moderate, and severe AD stages that were active at rest. Using the Group ICA Toolbox, these regions were retrieved using independent component analysis. Experiments are performed using an 80-20 split, where 80% of the data are used for model training and 20% are used for testing the models. A 10-fold cross-validation is used in this study.

### 3.1. Affected Brain Region Identification

In the initial phase of data analysis, the application of group-independent component analysis (ICA) is employed on fMRI data to extract insights from mixed data. The objective is to precisely identify the distinct brain regions impacted at various stages of AD. ICA, also referred to as the blind source separation (BSS) method, serves as a powerful tool for revealing hidden sources or factors that contribute to data, including those in fMRI. This technique has found extensive use in uncovering independent sources underlying complex information. In this study, by utilizing the ICA approach, a total of 20 brain regions are discerned across the four AD stages, each of which is associated with the disease progression within the dataset of 18 subjects.

Following data preprocessing, we used the Group Independent Component Analysis Toolbox (GIFT) to pinpoint the areas of the brain in people with MCI, including mild, moderate, and severe AD, that were active when they were at rest. These regions were recovered using independent component analysis (ICA), and GIFT provided peak coordinates for each component. Only the active brain areas are identified by GIFT; the precise name of each given region is not provided. Only the peak MNI coordinate of that specific location is provided. The GIFT toolbox’s MNI coordinates and anatomical automated labeling (aal. nii) were utilized to determine the name of a specific region. We identify the particular region by using MRIcron and peak coordinates in the MNI field.

MCI affected brain areas including the cuneus, precuneus, parahippocampal, frontal middle gyrus, putamen, and frontal superior gyrus. The calcarine cortex, frontal superior medial gyrus, frontal middle gyrus, and cuneus are the brain areas that are moderately affected. The calcarine cortex, precuneus, parahippocampal, frontal superior medial, frontal superior, and middle temporal gyri are affected brain areas in the severe stage of AD.

Affected brain regions in MCI include the cuneus, precuneus, parahippocampal, frontal middle gyrus, putamen, and frontal superior gyrus. Affected brain regions at the moderate stage are the calcarine cortex, frontal superior medial gyrus, frontal middle gyrus, and cuneus. Affected brain regions at the severe AD stage are the calcarine cortex, precuneus, parahippocampal, frontal superior medial gyrus, frontal superior gyrus, and middle temporal gyrus.

**Figure 2:**
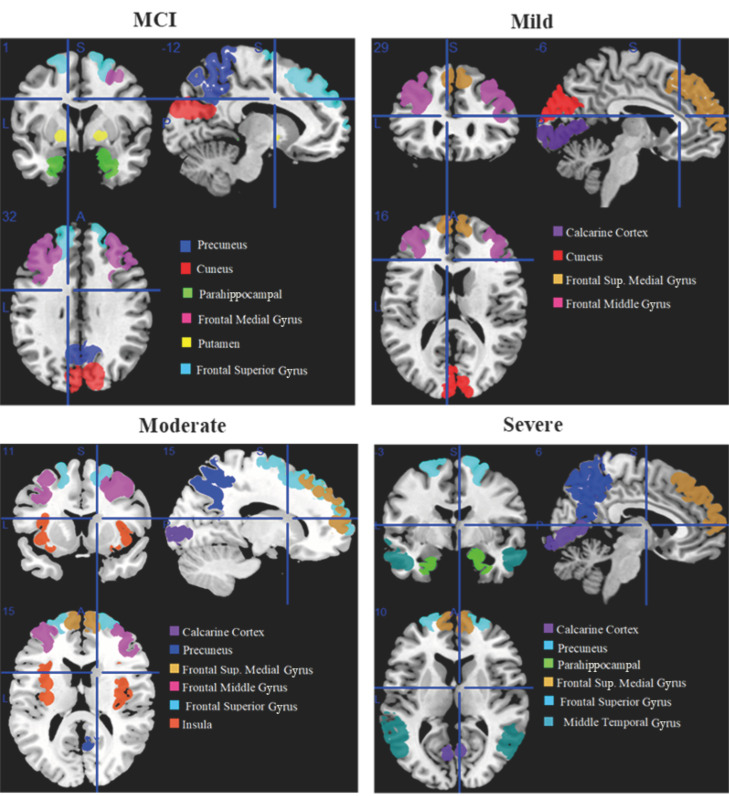
Orthogonal view of affected regions of the brain with MCI, including mild, moderate, and severe Alzheimer’s stage.

Figure 2 shows the brain regions that are active at rest in the early stages of MCI, including mild, moderate, and severe stages of AD, shown from an orthogonal perspective. Each region is assigned a different color, and the legends located at the bottom right of the image indicate the corresponding names of the brain regions. This orthogonal view is manually generated using features of MRICron.

### 3.2. Voxel Data Extraction

The voxel data for the appropriate brain areas were extracted at each stage using SPM12 when the brain regions were identified using ICA. Subsequently, the data analysis phase entailed the implementation of the general linear method (GLM). This technique enabled the extraction of voxels from the previously identified brain regions. Voxels, indicative of volume measurements within imaged structures, were used to pinpoint specific areas of interest. The activations within these identified brain regions displayed unique patterns across each AD stage. The corresponding numerical values representing these activations were meticulously recorded and are provided in tabular format within the paper.

First, we computed the mean image to aggregate the data from each subject. In other words, the 105 photos of each patient were added together. Then, using the labels provided in SPM12, the first level analysis was carried out and voxel data of specific regions were collected. The following specifications must first be given in order to extract data.

#### 3.2.1. Model Specification

**Figure 3:**
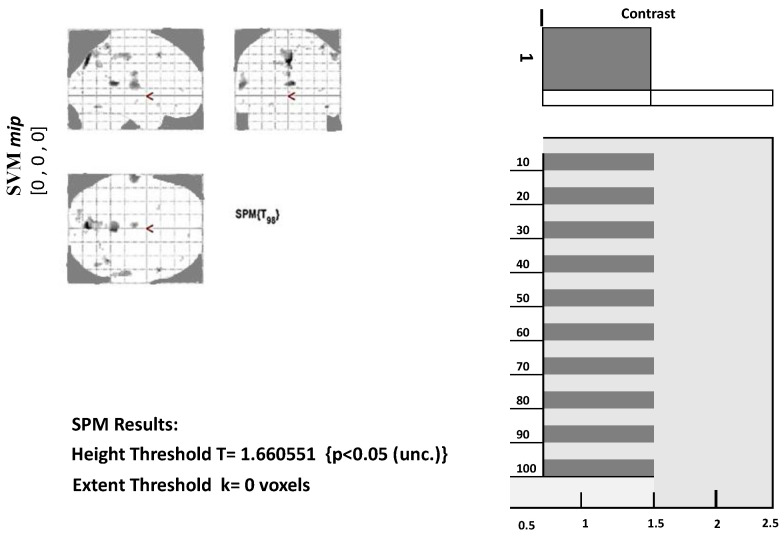
Glass brain view of Alzheimer’s disease brain at MCI-SPM output.

Before further analyzing fMRI data, the design matrix must be specified first. Several choices can be made depending on the conditions in the experiment. In the model specification, we need to set the output directory, data to be analyzed, inter-scan interval, scans per session, number of conditions, and the onset value. These values require a fixed model to run. The model specification will generate a file with the name spm.mat in the specified directory that will be used for further analysis. Figure 3 below shows the design matrix of a single AD patient at stage 1. Since the data were in a resting state, only one condition was set. The grey represents when the condition was on, and the white represents when the condition was set off. Similar design matrices were constructed at the second, third, and fourth stages for voxel data extraction.

#### 3.2.2. Model Estimation

The next step is to perform model estimation when the model is specified for the analysis. This is accomplished by selecting the spm.mat file from the directory. After execution, it will display a summary of the design matrix and parameters.

#### 3.2.3. Displaying Results

We first need to define a contrast to view the results from model estimation. Contrast may be set according to conditions involved in the experiment. In determining contrast, use 1 for the most active condition, a negative number for the less active condition, and 0 for the condition to be ignored. Next, we need to set a threshold for the set mask. The smaller the threshold value, the more significant the voxel will appear. We also need to place an extended threshold value, which means the minimum number of voxels in each cluster. Figure 3 below displays a glass brain view in which activated clusters/brain regions are marked with grey and black dots.

Along with the glass brain view, the *p*-value for the highly significant voxels of each cluster can also be obtained. Also, for each voxel, we can extract its values, as given in Table 3. Table 3 shows the number of voxels of 22 brain regions. The first level analysis was carried out, and voxel data for the targeted regions were extracted using the labels from SPM12.

The process of choosing particular brain regions or voxels of interest in neuroimaging studies is driven by a combination of scientific rationale and practical considerations. Researchers make these selections based on the specific hypotheses they aim to test, the existing body of knowledge in the field, and the underlying neurobiology of the condition under investigation. This process is integral to the study’s success, influencing the clarity of results and the ability to draw meaningful conclusions.

Table 4 presents the results attained by machine learning algorithms that were considered for classification. Considering the presented results, the overall performance of AdaBoost is better than all other models, with an average accuracy of 96.36% and precision, recall, and F1 scores of 96.50%, 95.50%, and 96.5%, respectively, followed closely by RF, with accuracy, precision, recall, and F1 scores of 93.27%, 95%, 93.5%, and 94%, respectively.

**Figure 4:**
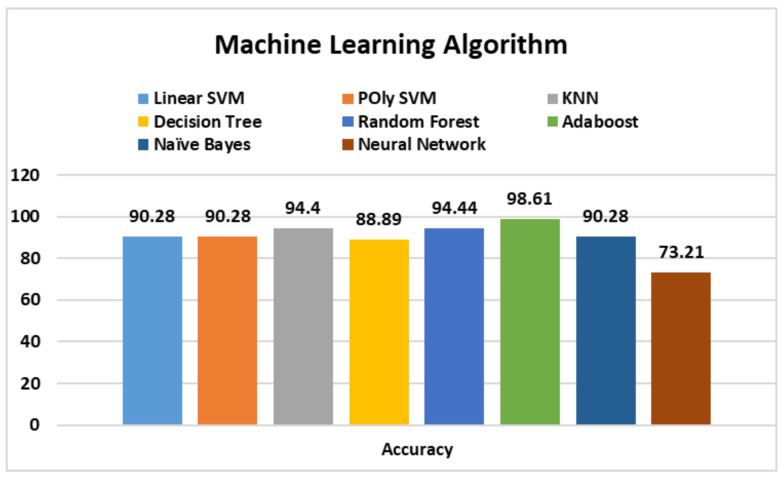
Accuracy comparison of machine learning models.

Figure 4 given above presents the comparative accuracy values. From the presented results, it can be seen that Adaboost and RF dominate other implemented machine learning algorithms. Therefore, the AdaBoost method is superior to the implemented machine learning algorithms.

### 3.3. Performance Comparison with Existing Studies

A performance analysis is also carried out to compare the performance of this study’s results with existing studies. For this purpose, [23,32,33,34,35] are selected because these studies also perform Alzheimer’s disease detection using the same dataset. These studies employed various machine learning models like SVM, AdaBoost, KNN, RF, etc. Table 5 shows the comparison of the results regarding reported accuracy. It can be observed that the results obtained in the current study are superior to those in the existing studies.

### 3.4. Relevance to Research Objectives

The brain regions or voxels chosen for analysis are often directly related to the research objectives. Researchers target areas known to be functionally or structurally linked to the cognitive processes or behaviors they intend to explore. For example, in studies of mood disorders, the amygdala and prefrontal cortex might be of particular interest due to their roles in emotional regulation.

#### 3.4.1. Prior Research

Existing literature plays a vital role in guiding the selection of brain regions. This study is built on previous studies that have identified specific regions as crucial for certain functions or implicated in the disorder being studied. This iterative process helps to advance knowledge and build a cohesive understanding of the brain’s complexities.

#### 3.4.2. Neurobiological Significance

Selections are often grounded in the known neurobiological relevance of specific regions. If a study pertains to sensory processing, brain areas associated with sensory modalities are natural choices. This ensures that the analysis aligns with the underlying neural processes.

### 3.5. Practical Considerations

- Statistical Considerations: Focusing on specific regions mitigates the multiple comparisons problem, reducing the risk of false positives. By narrowing the scope of analysis, researchers enhance the statistical power of their findings.
- Data Availability: Practical constraints, such as the scope of available imaging data or the research question’s focus, might dictate which regions are feasible to analyze.
- Analysis Complexity: Targeting specific regions simplifies the analysis process and aids in result interpretation. Analyzing a vast number of regions can lead to overfitting and complicate the interpretation of findings.
- Interpretation Clarity: Concentrating on selected brain regions enhances the interpretability of results. Researchers can provide more precise explanations and insights, facilitating a deeper understanding of the study’s implications.

### 3.6. Impact on Results

The decision to narrow the focus to specific brain regions has substantial ramifications for the study’s outcomes:

- Localization Precision: By concentrating on certain regions, researchers achieve higher spatial resolution, enabling a more accurate understanding of the neural underpinnings of behavior or cognitive functions.
- Sensitivity and Specificity: Focusing on particular regions can heighten the sensitivity to detect subtle effects within those areas. However, this specificity might reduce the model’s ability to detect effects in other regions.
- Generalizability: While the selected regions provide detailed insights, the findings might not be universally applicable to the entire brain or broader neural networks.
- Potential Insights Missed: Overly specific region selection could overlook relevant interactions or compensatory mechanisms occurring in other brain areas.
- Interpretational Bias: The choice of regions could introduce bias, influencing the interpretation and presentation of results.

In summary, the process of selecting specific brain regions or voxels of interest is a carefully considered decision that balances scientific intent, statistical power, and practical feasibility. These selections play a pivotal role in determining the study’s outcomes, influencing the accuracy of localization, sensitivity to effects, generalizability, and potential insights gleaned from the analysis.

### 3.7. Limitations and Future Work

While leveraging identified brain regions and their classification for AD, diagnosis, and prognosis offers promising avenues, there exist important limitations that call for consideration. The translation of intricate neuroimaging data into meaningful clinical insights remains a challenge, necessitating robust interdisciplinary cooperation among clinicians, data scientists, and neuroscientists. Moreover, the variability intrinsic to brain anatomy and imaging techniques introduces potential disparities that can impact the reliability of findings across studies.

In the pursuit of clinical applicability, bridging the gap between research discoveries and practical healthcare implementation becomes paramount. Developing user-friendly tools and decision support systems that harness brain region classification could empower clinicians with actionable insights for patient care. Additionally, the potential emergence of interventions targeting specific brain regions could redefine treatment strategies, potentially revolutionizing patient outcomes.

In essence, while the integration of brain region identification and classification holds immense potential, addressing challenges surrounding data interpretation, variability, and ethical considerations remains crucial. The trajectory of AD research will likely entail embracing advanced methodologies, adopting personalized approaches, and fostering seamless interdisciplinary collaboration to steer the field toward enhanced diagnosis, prognosis, and transformative treatment interventions within clinical settings.

## 4. Discussion

A neurological encephalopathy that is progressive and incurable is AD. The brain’s regional grey matter as well as areas especially linked to memory and cognitive behavior are damaged by AD. The veracity of these areas has been investigated and confirmed by numerous surveys. The hippocampus is the area of the brain that is affected by AD, according to [36]. The temporal lobe, insula, and posterior cingulate/precuneus are a few of the brain regions that show deterioration in grey and white matter in those migrating from MCI to AD [5]. Similar findings are made by [6], who discovered that neurodegeneration begins in the entorhinal cortex and hippocampus before spreading to other frontal, temporal, and parietal regions that are damaged by AD. The study in [7] reported decreased functional connectivity between the hippocampus and a number of brain regions, including the middle temporal gyrus, precuneus, inferior temporal cortex, medial frontal cortex, superior temporal cortex, and posterior cingulate gyrus. Additionally, they saw an improvement in the functional connection between the left hippocampus and the lateral prefrontal cortex.

By identifying the exact brain regions impacted at each stage of AD, the current study fills a gap in the body of prior literature. The GIFT Toolbox v4.0 of MATLAB was used to implement ICA. In order to determine whether brain regions related to working memory are impacted, Chatterjee et al. [26] employed GIFT to run ICA on fMRI data in cases of schizophrenia. Each subject’s 13 ICs were taken into account. After ICs were identified, the associated brain region was marked using the automated anatomical labeling (AAL) atlas. The most frequent ICs were identified as the brain regions that experience schizophrenia issues in their conclusion. Similar directions are taken by this present study, which involved four stages, looking at fMRI data from each patient at MCI, stage 1, stage 2, and stage 3 of AD. Each patient’s fifteen (15) ICs were examined, and those without a recognizable pattern were eliminated. The AAL atlas in the mricron32 program was used to identify the matching brain regions for the remaining ICs, and the generated ICs were validated using related techniques.

Most studies are conducted on MRI data which are structural data. Deep learning algorithms are highly recommended to analyze structural data because they compute structural changes in brain structure more efficiently than machine learning algorithms. We used fMRI data for our research, which are functional data. Localizing disease is a complicated task in fMRI data. Utilizing cutting-edge neuroimaging modalities such as fMRI, researchers can meticulously examine the intricate structural and functional changes unfolding within the brain. This scrutiny unveils subtle alterations that might precede noticeable symptoms of AD. Focused analysis of specific brain regions, notably the hippocampus, and select cortical areas typically vulnerable to the disease, can unveil incipient atrophy and functional anomalies, thereby facilitating early detection.

A statistical algorithm such as ICA makes it easy to identify the location of each component/brain region, and the brain region name is then identified using the MRICron tool. After identifying regions, we use GLM to extract the voxel means, a numeric value of activation of brain regions at each stage of AD. Brain region activation detail is in numeric form and the detail of activation is not too large. For smaller amounts of data and for precise computation, machine learning algorithms are better than deep learning algorithms. For this reason, this study employed machine learning algorithms. We intend to apply ensemble models and other advanced variants of machine learning models in future work.

Data leakage is an important concern for studies focusing on Alzheimer’s disease and several causes can lead to data leakage, as pointed out by [37]. For example, not splitting the dataset at the subject level may lead to data leakage. Samples from the same subject may appear in training, testing, and validation sets. Similarly, data augmentation before train–test splits may lead to similar data appearing in different sets. Biased transfer learning and lack of an independent test set are also attributed to data leakage. This study considered the data from the same subject for different stages of Alzheimer’s, which poses a risk of data leakage. However, the data from subjects are split for each stage separately, which removes the risk of data leakage at the subject level. A train–test split is used to make an independent test set comprising 20% of the data to avoid data leakage at this stage. No use of transfer learning and data augmentation also reduces the risk of data leakage in this study. K-fold cross-validation further corroborates the validity of the results.

## 5. Conclusions

This study extracted the voxel data of the identified brain regions at each stage using SPM12. The affected regions were deemed to be confirmed with a correlation value greater than/equal to 0.5. Secondly, the study affirmed some new regions that have not been reported to date in the literature related to working with brain regions during AD. Thus, this work may open a new path for researchers and may be a significant part of new studies in the future. It should be emphasized that some of the regions we found have already been mentioned in earlier research, although those studies were unable to identify the specific stage to which the regions belong. Second, when it comes to treating brain regions during AD, our investigation confirmed some new regions that have not yet been mentioned in the literature. As a result, this research could pave the way for future research in the field and contribute significantly.

Alzheimer’s patients are classified using fMRI scans, which are also used to examine brain areas linked to the condition. These images contain voxel data that are collected and processed. A multi-class classification framework was also established for the classification of specific locations linked to AD in addition to identifying the numerous brain areas that are impacted in cases of AD. Adaboost has proven to be the method with the highest level of accuracy. This strategy’s application yields prompt and accurate results. KNN and AdaBoost are strong algorithms that successfully handle classification issues, making them appropriate for this investigation. This technique makes it feasible to identify Alzheimer’s disease early, which enables quick treatment during the early stages and lowers the risk of severe consequences.

## Figures and Tables

**Table 1:** Number of samples of the dataset at each stage.

Stages	Samples	Volume
Stage 1	1890	105
Stage 2	1890	105
Stage 3	1890	105
Stage 4	1890	105
Total Samples	7560	-

**Table 2:** Summary of hyperparameters used in this study.

Model	Hyperparameters
AdaBoost	max_depth = 2, n_estimators = 300, learning_rate = 1.0
Decision Tree	Criterion = entropy, max_depth = 5, random_state = 0
KNN	n_neighbors = 5, metric=Minkowski, *p* = 1
Linear SVM	kernel=linear, C = 0.025, probability = true
RBF SVM	kernel = ploy, degree = 3, C = 1, probability = true
Neural Network	random_state = 1, max_iter = 300, probability = true
Random Forest	m_estimators = 1, max_features = none, bootstrap = false, random_state = 1

**Table 3:** Number of voxels extracted at each stage of Alzheimer’s disease.

Brain Region	# of Voxels at MCI	# of Voxels at Mild	# of Voxels at Moderate	# of Voxels at Severe
Calcarine_cortex_left	426	543	481	617
Calcarine_cortex_right	398	480	445	561
cuneus_left	320	597	664	556
Cuneus_right	347	645	622	556
frontal_middle_left	903	1451	1323	1162
frontal_middle_right	1173	1296	1442	1099
Hippocampus_left	159	319	162	312
Hippocampus_right	191	282	222	357
insula_left	822	1008	286	1132
insula_right	931	957	288	1039
middle_temporal_left	166	365	211	432
middle_temporal_right	166	271	376	124
parahippocampal_left	153	173	162	103
parahippocampal_right	153	96	81	96
precuneus_left	1429	1917	2045	1610
precuneus_right	1364	1838	1915	1524
putamen_left	553	548	160	678
putamen_right	553	556	92	561
superior_frontal_left	583	785	936	375
superior_frontal_right	583	741	1111	267
superior_frontal_medial_left	607	711	72	455
superior_frontal_medial_right	665	742	161	480

**Table 4:** Classification results for machine learning models.

Model	80-20 Split Results (Avg. %)				10-Fold Results (Avg. %)			
	**Accuracy**	**Precision**	**Recall**	**F1 Score**	**Accuracy**	**Precision**	**Recall**	**F1 Score**
Linear SVM	90.28	91.00	90.00	90.00	90.76	92.00	86.00	87.00
Poly SVM	90.28	91.00	90.00	90.00	90.70	88.00	84.00	84.00
KNN	94.40	96.00	94.00	95.00	93.80	91.00	86.00	88.00
Decision Tree	88.89	90.00	89.00	89.00	87.41	89.00	92.00	87.00
Random Forest	94.44	94.00	94.00	94.00	92.10	96.00	93.00	94.00
AdaBoost	98.61	99.00	98.00	98.00	94.11	94.00	93.00	95.00
Naïve Bayes	90.28	92.00	90.00	90.00	94.90	94.00	98.00	95.00
Neural Network	73.21	74.00	74.00	73.00	72.70	74.00	73.00	69.00

**Table 5:** Comparison with existing works on Alzheimer’s disease detection.

Ref.	Approach	Reported Accuracy	This Study
[23]	Employed SVM algorithm to make binary classification among various stages of AD.	78.22%	90.28%
[32]	Author was able to improve the classification accuracy of earlier studies on the early identification of AD by using AdaBoost. The three target classes used in the study were normal (CN), mild cognitive impairment (MCI), and AD. The study used the ADNI clinical dataset.	92.70%%	98.60%
[33]	The paper utilized machine learning to create a multistage classifier for Alzheimer’s disease, incorporating NB classifier, SVM, and KNN models. The study aimed at achieving improved and efficient classification of Alzheimer’s disease.	90.47%	94.40%
[34]	The paper utilizes SVM and KNN algorithms that rely on psychological parameters such as age, number of visits, MMSE, and education to make predictions about Alzheimer’s disease.	SVM: 85%, KNN: 83%	SVM: 90.2%, KNN: 88.80%
[35]	Author trained RF and other machine learning models in this study using regional volumes from 2250 brain MRIs. The study’s dataset, which comprised 687 NC, 1094 people with MCI, and 469 people with AD, was taken from the ADNI database.	90.00%	94.40%

## Data Availability

Alzheimer’s Disease Neuroimaging Initiative’s ADNI dataset is available at: https://adni.loni.usc.edu.
